# Three-Dimensional *In Vitro* Models of Granuloma to Study Bacteria-Host Interactions, Drug-Susceptibility, and Resuscitation of Dormant Mycobacteria

**DOI:** 10.1155/2014/623856

**Published:** 2014-05-21

**Authors:** Liam E. Fitzgerald, Naiara Abendaño, Ramon A. Juste, Marta Alonso-Hearn

**Affiliations:** Department of Animal Health, Basque Institute for Agricultural Research and Development, NEIKER-Tecnalia, Technological Park of Bizkaia, Derio, 48160 Bizkaia, Spain

## Abstract

*Mycobacterium tuberculosis*, *Mycobacterium leprae*, *Mycobacterium bovis,* and *Mycobacterium avium* subsp. *paratuberculosis* can survive within host macrophages in a dormant state, encased within an organized aggregate of immune host cells called granuloma. Granulomas consist of uninfected macrophages, foamy macrophages, epithelioid cells, and T lymphocytes accumulated around infected macrophages. Within granulomas, activated macrophages can fuse to form multinucleated giant cells, also called giant Langhans cells. A rim of T lymphocytes surrounds the core, and a tight coat of fibroblast closes the structure. Several *in vivo* models have been used to study granuloma's structure and function, but recently developed *in vitro* models of granuloma show potential for closer observation of the early stages of host's responses to live mycobacteria. This paper reviews culture conditions that resulted in three-dimensional granulomas, formed by the adhesion of cell populations in peripheral blood mononuclear cells infected with mycobacteria. The similarities of these models to granulomas encountered in clinical specimens include cellular composition, granulomas' cytokine production, and cell surface antigens. A reliable *in vitro* dormancy model may serve as a useful platform to test whether drug candidates can kill dormant mycobacteria. Novel drugs that target dormancy-specific pathways may shorten the current long, difficult treatments necessary to cure mycobacterial diseases.

## 1. Zoonotic Diseases Caused by Mycobacteria

Zoonotic diseases are infectious diseases naturally transmissible between vertebrate animals and humans. More than 60% of all human pathogens are zoonotic, including 75% of the past decade's emerging pathogens. Pathogens circulating human and animal populations very often risk public health as well as animal health, so both the animal and human health sectors are responsible for their detection, prevention, and control according to the “one world-one health” concept (WHO, OIE, FAO). However, the vast majority of zoonoses are labeled neglected zoonotic diseases (NZDs) because they are not prioritized by national and international health systems. The impact of NZDs is most severe in developing countries, as reliance on animals for food, transport, and farm work leads to continual close contact with animals in rural populations. Zoonotic diseases often go undiagnosed and untreated in these regions due to lacking public awareness and education on preventive measures and also due to the lack of political commitment and funding of veterinary and health services.

Around 15 species of pathogenic mycobacteria for human beings and/or animals are known and are leading health concerns.* M. tuberculosis* causes most cases of tuberculosis (TB) in humans and is the leading cause of mortality due to a single infectious agent among human adults in the world [1, 2]. In 2011, there were an estimated 8.7 million new cases, 1.4 million deaths, and about 2 billion latent infections caused by* M. tuberculosis* [3]. Animal infection with* M. tuberculosis*, while being uncommon, has been described among nonhuman primates and several other species such as birds, elephants, and other mammals, after prolonged close contact with humans. However, the overall prevalence of disease in these susceptible species is low and documented transmissions of* M. tuberculosis* between animals and humans are uncommon [4–6]. Along with* M. tuberculosis*,* M. bovis* causes TB in humans, though far less commonly than* M. tuberculosis *[7, 8]. As a result of milk pasteurization and* M. bovis* eradication programs in most industrialized countries, zoonotic transmission of* M. bovis* through domestic livestock is now rare. In contrast, similar eradication programs have not been conducted for wild animals. Consequently,* M. bovis* is widely prevalent in cattle and wild animals and is responsible for $3 billion global economic losses a year [9]. Recently, two people were identified as having caught TB from cats but the risk of cat-to-human bovine TB transmission is very low [10]. Paratuberculosis or Johne's disease (JD) causes major economic losses to the global dairy industry due to lower milk production, reduced slaughter value, increased premature culling, and increased calving intervals [11]. Total losses due to JD in US dairy herds were estimated at $200–250 million annually, and the prevalence of JD at the herd level is probably much higher than 50% in most countries with a significant dairy industry [12].* M. avium *subsp* paratuberculosis (Map)*, the causal agent of JD in domestic ruminants and wildlife animal species, may also have human health significance as a causal or exacerbating agent in human Crohn's disease (CD), a chronic inflammatory bowel disease characterized by transmural inflammation and granuloma formation. Evidences that* Map *may be associated with CD in humans include similarity between the clinical signs of CD in humans and those found in animals with paratuberculosis; detection of* Map* in feces, intestinal tissues, breast milk, macrophages, and peripheral blood of patients with CD; association between* Map* DNA in blood and cellular and humoral immune responses in CD; and anti-*Map *antibiotic therapy resulting in reduction of bacteremia and remission, or substantial improvement in disease condition in many patients [13–17]. In addition, meta-analysis studies have confirmed an association of* Map* with CD [18, 19].

*M. leprae *has been eliminated from most countries due to an aggressive push by the WHO in the 1980s and 1990s, but it continues as a public health problem in tropical and semitropical countries. A total of 219,075 new cases of leprosy were reported in 2011 according to the WHO [20]. Nonhuman reservoirs of* M. leprae* exist in monkeys and armadillos and three case-control studies have shown contact with armadillos to be a significant risk factor for leprosy in the United States [21, 22]. Naturally acquired murine leprosy has been observed in rats, mice, and cats but not in humans or any other species. Thus, in contrast to human leprosy, murine leprosy is not a zoonosis [23].

## 2. Mycobacterial Infections Induce Granuloma Formation

A defining aspect of the immune response against mycobacteria is the formation of organized cellular aggregates called granulomas. Following internalization of pathogenic mycobacteria by host macrophages, activated lymphocytes and infected macrophages secrete cytokines and chemokines which trigger an inflammatory response including recruitment of blood and tissue macrophages and T-lymphocytes to the infection site. This accumulation of cells around infected macrophages is called a granuloma. Granulomas consist of lipid-loaded macrophages (foamy macrophages), epithelioid cells (differentiated macrophages) with a larger cytoplasm and interdigitated cell membranes, and multinucleated giant cells (Langhans cells) [24–26]. T and B lymphocytes surround the granuloma core, and a tight coat of fibroblasts and collagen closes the structure, now a complete granuloma [27, 28]. Mycobacteria-infected macrophages are sequestered inside the granuloma's hypoxic environment. The accumulation of intracytoplasmic lipid inclusions, the loss of acid fastness, the development of resistance to the antibiotic rifampicin, and the arrest of bacterial multiplication have all been described as the major hallmarks of dormant bacteria within granulomas [29–33].

Granulomas have long been considered as a natural defensive mechanism meant to contain the host immune response and mycobacteria in a localized area, preventing bacterial spread to surrounding healthy tissues or other organs [34]. Complete eradication of the bacteria does not occur inside granulomas because pathogenic mycobacteria have developed their own strategies to evade the host immune response and persist within the granuloma in a dormant state for decades while continual replenishment of white blood cells keeps the granuloma [35]. If the host's immunity is weakened and/or suppressed, the dormant mycobacteria may reactivate and escape from the granuloma and form lesions in other parts of the tissue [36]. For instance,* M. tuberculosis *can persist for decades within granulomas and may reactivate if the host's immunity is weakened due to an HIV infection, diabetes, cancer, malnutrition, aging, or host genetic factors [37, 38]. However, the metabolic and replicative state of the bacteria in the granulomatous lesions of asymptomatic humans remains a controversial issue [39]. Although granuloma formation has long been thought to be a host-driven process to contain infection, recent studies indicate that granulomas contribute to early bacterial growth and that pathogenic mycobacteria exploit the granuloma for local expansion and systemic dissemination [40].

As mentioned before, the secretion of various cytokines and chemokines is an essential element in the early immune response against mycobacteria infection and in the early steps of granuloma formation [41]. Following internalization of mycobacteria by host macrophages, activated macrophages release IL-8, a powerful chemoattractant for T lymphocytes, which will surround the granuloma structure [42]. Within the granuloma, activated and recruited T-lymphocytes secrete interferon (IFN-*γ*), which in turn activates additional macrophages [43]. Tumor necrosis factor-*α* (TNF-*α*) is produced by infected macrophages and plays an important role in the accumulation and differentiation of macrophages into the highly bactericidal epithelioid cells present in granulomas [44]. TNF-*α* is also implicated in the maintenance of the granuloma structure by maintaining cellular recruitment [45–47]. Mycobacterial-infected macrophages also release large quantities of IL-6, which plays a role differentiating activated macrophages to multinucleated giant cells [48].

## 3. *In Vivo* and* In Vitro* Granuloma Models

Analysis of host-pathogen interactions inside granulomas is crucial to understand the pathogenesis of mycobacterial infections. Since the access to granulomas in human biopsy samples is often limited, animal models of TB granulomatous inflammation have been developed, including use of monkeys, mice, rabbits, and guinea pigs [49]. The most extensively used animal model, the mouse, is not a natural host of* M. tuberculosis*. Although granulomas can be seen in mice, they are small, have a different cellular organization than in humans with absence of necrotic areas and multinucleated giant cells, and are unable to establish latency [50, 51]. Rabbits are susceptible to* M. bovis *infection and guinea pigs are susceptible to* M. tuberculosis*. Although these animal models develop classical granulomas, they lack true latency, and the immune response generated in these* in vivo *models is likely quite different than the cattle or human response [52, 53]. Nonhuman primate models more closely resemble the wide spectrum of granulomas in human TB, and latency can be well established in these models. However, macaques are very expensive to maintain and difficult to handle and imply ethical considerations [54]. Cattle can be experimentally infected with* M. bovis* or* Map *but these experiments are expensive because they require several months of maintenance under BSL3 conditions for the induced granulomas to be large enough for study [55, 56]. Another common drawback of* in vivo* models is that they can only be used to study highly differentiated granulomas, preventing the analysis of the processes involved in their very early development [57].

In order to study the very first steps of the granuloma formation,* in vitro* hypoxic-induced, stress-induced, or granuloma models have been developed. The advantages of* in vitro* models include reduced cost, increased control, and that they can provide insights into host-mycobacteria interactions at early stages of granuloma formation.* In vitro* models mimic the conditions encountered by the bacteria within host granulomas (hypoxic-induced or stress-induced models) or the physiological granuloma (granuloma models). However, the granuloma constitutes a complex immune microenvironment highly affected by additional physiological signals (i.e., growth factors and cytokines) which are exclusively produced in infected tissues. As consequence, certain aspects of* in vivo* granulomas may be different or absent in* in vitro *models, including intragranulomatous necrosis, accumulation of fibrin and collagen, and presence and distribution of bacilli. Using* in vitro* models, important information has been achieved about granuloma cell differentiation as well as about cellular interactions and cell/bacteria interplay within granulomatous structures. In a hypoxic-induced environment,* M. tuberculosis* accumulates triacylglycerides (TAG) within intracytoplasmic inclusions (ILI) and enters into a nonreplicative state; upon reexposure to oxygen, the pool of TAG within ILI is drastically reduced and bacilli undergo regrowth [58]. Recently, Deb et al. developed a multistress dormancy model for* M. tuberculosis* and showed that* M. tuberculosis* exhibited all the hallmarks characteristics of dormancy [59]. Similarly, in lipid-loaded THP-1 derived macrophages,* M. tuberculosis* has been found to accumulate TAG and stop replication [33]. Although most stress-induced models of* M. tuberculosis *dormancy are able to induce a dormant state of the bacteria, they have been unable to demonstrate resuscitation under conditions that mimic immune suppression. To achieve this goal, three-dimensional* in vitro* models of granulomas have been developed, which may more closely resemble not only dormancy but also resuscitation of the bacteria under conditions that mimic immune suppression (Table 1). Therefore, granuloma models can be used to study both latent and active stages of infection. Three-dimensional granuloma formation occurs* in vitro* only when infected peripheral blood mononuclear cells (PBMCs) are cultured under conditions that inhibit surface contact.

## 4. Three-Dimensional* In Vitro* Models of* M. tuberculosis* Granulomas

Several reports have described three-dimensional models of* M. tuberculosis* granuloma. The first model of early granuloma formation using PBMCs infected with* M. tuberculosis* or* M. bovis *was reported by Seitzer and Gerdes [60]. PBMCs were seeded into agarose-coated wells of 96-well plates at a density of 6 × 10^5^ in 200 *μ*L of supplemented RPMI-1640 medium and infected with* M. tuberculosis* strain H37Rv or* M. bovis* BCG (bacille Calmette-Guerin) at MOI (bacteria/cell) of 1 : 150, 1 : 85, 1 : 42, and 1 : 4. After 4 days of incubation only the MOI 1 : 150 produced aggregates. Infection at higher MOI did not result in spheroid formation but rather in several very small aggregates and a dramatic increase in the number of dead cells. The authors verified this model as comparable to* in vivo* granulomas by assessing the aggregate cell types and cell differentiation through histology and immunostaining. By histological analysis, they observed that the generated heterospheroids shared many phenotypical characteristics of granulomas, such as three-dimensional aggregation of monocytes, B cells and T cells and the presence of macrophages, multinucleated giant cells, and necrotic areas. By immunostaining, the presence of disperse CD14^+^ monocytes, CD163^+^ macrophages and CD3^+^ T cells was demonstrated in the granulomas together with a ring of CD19^+^ B cells in the peripheral zone.

Birkness et al. observed the formation of small, round, granuloma-like aggregates by combining human PBMCs, autologous macrophages, and* M. tuberculosis *in ultralow attachment tissue culture plates [61]. When the MOI was 1 : 400 or 1 : 4000, and when nonadherent PBMCs were added 2 and 5 days after infection to simulate the natural recruitment of additional lymphocytes at the infection site, small aggregates consisting of multinucleate-giant cells, epithelioid macrophages, and T-lymphocytes were observed. Immunostaining showed CD68^+^ epithelioid macrophages and small numbers of CD3^+^ T-lymphocytes that resembled the granulomas seen in clinical specimens. Acid-fast bacteria were observed between and possibly within the cells composing the granulomas. In addition to looking at the cellular differentiation of the resulting aggregates, Birkness et al. examined the immunological response of the host cells as these aggregates formed. Supernatants from the infected cells were collected at different time points, analyzed by multiplexed cytokine bead-based assay, and found to contain IL-6, IL-8, IFN-*γ*, and TNF-*α*. Secretion of detectable quantities of these proteins over the first 9 days following infection required a MOI of 1 : 4–1 : 40. Finally, it was found that the additions of IL2, IFN-*γ*, and/or TNF-*α*, which are known to play a role in cell recruitment and granuloma formation, greatly enhanced the formation of aggregates.

The three-dimensional models described above were unable to demonstrate mycobacterial dormancy and resuscitation inside the three-dimensional generated granulomas. Recently, Kapoor et al. developed a three-dimensional* in vitro* granuloma model in which* M. tuberculosis* goes into dormancy and subsequently resuscitates under conditions that mimic weakening of the immune system [62]. Human PBMCs were mixed at room temperature with an extracellular matrix (ECM) at 5 × 10^5^ cells/50 *μ*L/well of a 96-well plate.* M. tuberculosis *H37Rv was added to the ECM at MOI 1 : 10. Samples were allowed to set by incubating at 37°C for 45 min. RPMI containing 20% of human serum was added and the samples were incubated for 8 days in a 37°C incubator. Infected PBMCs formed microgranulomas, observed by the aggregation of lymphocytes around infected macrophages and the presence of multinucleated giant cells. The ECM was prepared by mixing 0.95 mL of collagen solution (Purecol, USA), 50 *μ*L 10 × DPBS (Lonza, US), 4 *μ*L fibronectin (BD Biosciences, USA), and 10 *μ*L 1N NaOH per mL of matrix. In this* in vitro *model, IFN-*γ*, TNF-*α*, subunit-*β* of IL12 (IL-12p40), and interferon-*γ* induced protein 10 (IP-10) were induced by* M. tuberculosis* infection and detected in the culture supernatants from day 8 granulomas. In this model,* M. tuberculosis* goes into a dormant state, demonstrated by loss of acid fastness, accumulation of lipid bodies, development of rifampicin tolerance, and gene expression changes. Treating granulomas with an immunosuppressant anti-TNF-*α* monoclonal antibody caused reactivation of dormant* M. tuberculosis*.

## 5. Three-Dimensional* In Vitro *Models of* M. leprae *Granulomas

Wang et al. described an* in vitro* model of* M. leprae* granuloma. Monocytes-derived macrophages were infected in a 24 well-tissue culture plate with* M. leprae* at MOI 50 : 1 [63]. Autologous human PBMCs were added after 24 hours and the cells cultured in RPMI containing 20% fetal calf serum (FCS) at 35°C for additional 9 days. Phase-contrast, electron, and confocal microscopy revealed the presence of monocytes around macrophages infected with* M. leprae* and the formation of multinucleated giant cells and epithelioid cells, both of which resemble the cells seen in histopathological granuloma specimens of tuberculoid leprosy lesions. Patterns of cell antigen expression and cytokine production appeared consistent with those observed in* M. leprae* lesions.* M. leprae* was seen within multinucleated giant cells, and the bacteria remained metabolically active, demonstrated by CO_2_ production which fell insignificantly.

## 6. Three-Dimensional* In Vitro *Models of* M. bovis *Granuloma

The model of Puissegur et al. used human PBMCs and artificial beads coated with* M. tuberculosis* antigens or live* M. bovis* BCG to form aggregates displaying morphological characteristics and cellular differentiation very similar to natural granulomas [64].* M. bovis *BCG and human PBMCs were incubated at 37°C in a 5% CO_2_ atmosphere for 15 days. The granulomas were collected at different times of incubation and prepared for scanning electron microscopy. At six hours, monocytes were gathering around the bacteria. After 4 to 5 days, lymphocytes began to be recruited around the structures, which then kept on growing with cell recruitment until incubation stopped. To better assess the internal structure of these in* vitro* granulomas, day 9 BCG granulomas were collected, fixed, and embedded in Epon-araldite resin. Transverse sections (0.5 *μ*m) of the granulomas were then stained and analysed by transmission electron microscopy. Activated lymphocytes presenting tight contacts with activated macrophages and multinucleated cells were visible in the centre of a BCG-induced granuloma.

## 7. Three-Dimensional* In Vitro* Models of* M. avium *Subsp*. paratuberculosis *Granulomas

**Figure 1:**
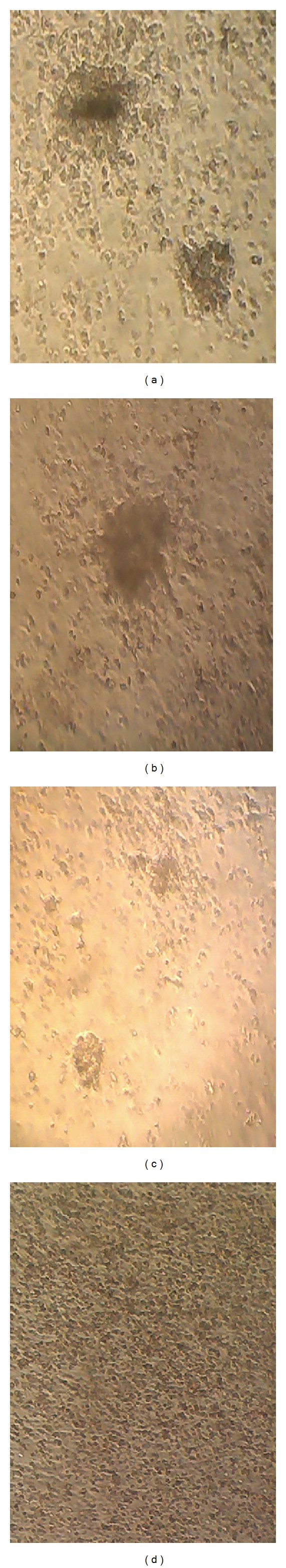
Phase contrast images showing the presence of* in vitro *granuloma-like aggregates after the infection of bovine PBMCs with the bovine K10 strain of* M. avium* subsp.* paratuberculosis *at MOI (bacteria : cells) of 1 : 16 (a) and 1 : 8 ((b) and (c)). Original magnification is 10x. Uninfected cells show an absence of granuloma formation (d).

Birkness et al. mention only briefly in their paper that* in vitro* granulomas also formed in response to infection with other viable mycobacteria including* Map *and that the aggregates were macroscopically similar to the granulomas formed from* M. tuberculosis* infection but did not show any images of these specific aggregates [61]. We have recently infected bovine PBMCs placed in a collagen matrix with a bovine isolate of* Map* at a MOI = 1 : 8 or 1 : 16. Our extracellular matrix was prepared according to Kapoor et al. with some minor modifications [62]. Plates were incubated for 10 days and examined for granuloma formation by phase contrast microcopy using an inverted microscope.  Figure 1 shows the morphological characterization of the three-dimensional granuloma-like aggregates formed by primary bovine PBMCs infected with* Map*. Aggregates started to form 2-3 days after infection and their relative number increased with time. In addition, the aggregates become larger and more clearly defined with time. Uninfected samples from the same donor did not form these aggregates indicating aggregation is a response to* Map* infection. Fixation and paraffin-embedding of the aggregates are currently being performed for histological examination of the microgranulomas' ultrastructure.

## 8. Conclusions

The four mycobacterial diseases discussed in this paper cause chronic granulomatous inflammation and the treatment for each is limited by the presence of dormant bacteria within host granulomas. Granulomas have long been studied using* in vivo *models, but recent* in vitro *models may allow closer analysis of the host-pathogen interactions involved, making study of these models relevant to treatment. Our review suggests that three-dimensional* in vitro* models are potentially comparable to granulomas observed in clinical specimens with respect to the cellular components involved, patterns of cell surface expression, cytokine and chemokine secretion, development of mycobacterial dormancy, and resuscitation under conditions that suppress the host immune response. These models could potentially provide insights into host-mycobacteria interactions at stages of granuloma formation too early to address with animal models. Three-dimensional* in vitro* models of granuloma formation may be useful to (i) understand what factors or molecules play a role in granuloma formation and in its continued integrity, (ii) evaluate the granuloma-inducing activity of particular antigens or attenuated mutants, (iii) provide a platform for testing vaccine and drug candidates against dormant as well as active mycobacteria, and (iv) characterize the molecular interplay between mycobacteria and host cells within granuloma structures.

## Figures and Tables

**Table 1:** Three-dimensional *in vitro *granuloma models.

Bacteria	Cells	MOI (bacteria : cells)	Extracellular matrix	Growth medium	Days	Year	Reference
*M. tuberculosis *and* M. bovis *	Human PBMCs	1 : 150	Agarose coated 96-well tissue culture plates	RPMI-1640, 100 U/mL penicillin G, 100 *μ*g/mL streptomycin, and 10% FCS	7	2003	[60]

*M. tuberculosis *	Human PBMCs and autologous macrophages (1 : 1)	1 : 4–40 for host cell cytokine production	Ultralow attachment 24-well tissue culture plates	RPMI-1640, 10% human serum	9	2007	[61]

*M. tuberculosis *	Human PBMCs and autologous macrophages (1 : 1), extra nonadherent PBMCs added at 2 and 5 days	1 : 400–4000 for histology and immunostaining	Ultralow attachment 24-well tissue culture plates	RPMI-1640, 10% human serum	9	2007	[61]

*M. tuberculosis *	Human PBMCs	1 : 10	0.95 mL collagen solution, 50 *μ*L 10xDPBS, 4 *μ*L fibronectin, 10 *μ*L 1 N NaOH	RPMI-1640, 20% human serum	8	2013	[62]

*M. leprae *	Human macrophages and autologous PBMCs (1 : 5)	50 : 1	24-well tissue culture plates	RPMI-1640, 20% FCS	10	2013	[63]

*M. bovis *	Human PBMCs	1 : 10	24-well tissue culture plates	RPMI-1640, 10% human serum, 300 U/mL penicillin, 0.3 mg/mL streptomycin	15	2004	[64]

*M. avium *subsp* paratuberculosis *	Bovine PBMCs	1 : 8 and 1 : 16	0.95 mL bovine collagen solution, 50 *μ*L 10xDPBS, 4 *μ*L fibronectin (1 mg/mL), 10 *μ*L 1 N NaOH	RPMI-1640, 100 U/mL penicillin G, 100 *μ*g/mL streptomycin, 20% FBS	10	2014	Current report

FCS: fetal calf serum; PBMCs: peripheral blood mononuclear cells; DPBS: Dulbecco's phosphate-buffered saline; FBS: fetal bovine serum.
